# Solution Evolution Knowledge Service Based on Design Iteration in Strain Sensor Design

**DOI:** 10.3390/s23041931

**Published:** 2023-02-09

**Authors:** Kai Zhang, Wu Zhao, Qingjie Liu, Xin Guo, Miao Yu

**Affiliations:** 1 School of Mechanical Engineering, Sichuan University, Chengdu 610065, China; 2 Innovation Method and Creative Design Key Laboratory of Sichuan Province, Chengdu 610065, China; 3 Department of Aircraft Manufacturing, Sichuan Aerospace Vocational College, Chengdu 610100, China

**Keywords:** knowledge service, design iteration, solution evolution, nanofiber, strain sensor

## Abstract

Product design is a process of repeated iteration and gradual improvement, and knowledge push is one of the bottlenecks that needs to be solved to improve the product design level. With the increase in design complexity and iteration rounds, the existing knowledge application methods can hardly meet the needs of product design solution iteration and evolution. In order to better assist designers in acquiring and applying knowledge in the process of product design solution evolution, a knowledge service method for product design solution evolution based on the problem–strategy–solution (PSS) interaction iteration is proposed. The mapping and feedback process between design problems, design strategies, and design solutions are analyzed, a model of the solution evolution process based on design iteration is proposed, and a PSS-based product design solution evolution mechanism is established. On this basis, the product design solution evolution knowledge service dimension is built, and the solution evolution knowledge service model based on design iteration is established. The corresponding solution evolution function module is developed based on the pre-developed computer-aided product innovation design platform. The validity of the iterated-based design was proved in the technical innovation of nanofiber preparation and further application of strain sensors.

## 1. Introduction

Innovative design is an iterative and progressive refinement process that accompanies the iteration of the design process and the evolution of the design solution. When one wants to achieve a specific goal, but cannot immediately find a suitable path, the activity one engages in can be called problem solving [1]. Most designers consider problem solving to be a linear process. However, since design problems are not always well defined at the beginning of the design task, design problems can be ill defined, ill structured, and wicked [2]. The problem-solving activities are complex, involving the interweaving iteration and evolution of different design elements, and eventually evolving the design problem into a well-structured problem. Innovative design involves defining design problems and reconstructing them in the design process, which makes the whole design process dynamic and repetitive [3]. Therefore, it is significant to study effective innovative design processes and strategies to guide designers to solve design problems quickly.

Knowledge is an indispensable resource for product innovation design. Different design phases have different design needs and require different knowledge. The management and application of knowledge is a key factor for companies to achieve innovation and win competition [4,5,6,7]. The traditional product innovation solution model is limited to the specific form of the solution or problem. It represents the mapping relationship between function, behavior, and structure through a top-down unidirectional mapping process, which only solves the product function from a single perspective, imperfectly constructs the design space, and ignores the fact that the interaction relationship between workflow, information flow, knowledge flow, and other factors in the actual design process is the key to generate innovative solutions [8]. Others may focus only on the construction of the solution space, ignoring the synergistic iteration of the solution space and the problem space, and the flow of knowledge at different levels of abstraction. These studies make it difficult to assist designers in applying knowledge for better iteration, reconstruction, and evolution of design solutions by means of superposition and migration. Thus, product innovation design requires not only constructing problem space and solution space, but also guiding designers to use knowledge flexibly with creative thinking, overlaying multi-domain knowledge, applying innovative methods, and stimulating knowledge in the process of design retrospection and solution evolution.

It is considered that the product innovation design process is a complex project to solve the ill-structured problem, which is the result of the interrelated and synergistic evolution of design problem space and design solution space under the guidance of design thinking and design strategy. Understanding the law and pattern of the iterative evolution of product design solutions and constructing the correlation relationship between design problems, design strategies, and design knowledge in the process of solution evolution are the prerequisites for realizing knowledge-supported innovative design. Therefore, this paper aims to further explore the evolutionary mechanism of product innovation design and solve the problems of the iterative evolution mechanism of product design solutions and the mapping relationships between design problems, design strategies, and design knowledge in the evolution process. A new evolutionary model of product innovation design solutions is proposed in this paper. Within the framework of this model, the role and relationship of design problems, design solutions, and design strategies in the innovation solution process are studied. A workflow of product innovation solutions that conform to the design law and the cognitive law of the designer is formed so that the creativity of the designer can be given full play. The main contribution of this research is to establish the correlation between design problems, design strategies, and design knowledge, to provide a concrete model framework and theoretical methods for the iterative evolutionary process of product innovation design, which will provide knowledge service ideas for the multidisciplinary intersection of research work such as electromechanical device design, nanomaterials, and sensor development.

## 2. Related Works

**(1)**  **Research on conceptual design process models**

Conceptual design process models have been studied for decades, mainly in terms of the basic laws, principles, and processes of design, and as a theoretical basis for design methods, mainly used to standardize and support the process of using design methods. Jing et al. proposed a multi-interaction qualitative objective decision-making method of a product conceptual scheme based on non-cooperative and cooperative game theory to solve the fuzzy problem of constraints between product conceptual design objectives, and established a cooperative game decision-making model to screen out the optimal design scheme [9]. The conceptual design process of the systematic approach proposed by Pahl et al. includes phases of information definition, creation, evaluation, and decision, etc. [10], with the core of obtaining substructures through functional decomposition and principle solving, and finally obtaining a conceptual solution through combination and evaluation. Camelo et al. proposed a product conceptual design process model based on multiple relationships and interactivity to expand the search space of design schemes by expanding the relationships between design elements [11]. Chen et al. proposed a conceptual design process model of requirements–function–principle system, including clarification, synthesis, implementation, analysis, and prediction phases, through a normative definition of relevant design concepts [12]. Feng et al. proposed a conceptual design framework based on product genetic inheritance and recombination by comparing the relationship between biogenetic engineering and product principle scheme design [13]. Li et al. took the conceptual design process based on problem solving as a research category and divided it into multiple phases, such as problem definition, problem analysis, concept generation, scheme design, and scheme evaluation [14]. Wan et al. applied QFD, the object field model, conflict matrix, and the morphological analysis method to problem analysis and concept solving, and proposed a multi-method integrated product innovation strategy and design process [15]. Li et al. proposed an integrated conceptual design process model containing five phases and four mappings and used a mathematical language to describe the integration logic of this process model [16]. Kansei engineering correlates perceptual imagery with design features through the steps of cognitive vocabulary perceptual quantification, engineering scale transformation, and the integration of engineering design elements in an engineering approach [17]. Xu et al. proposed a drive–implication–matching model based on the constructive design theory, which completes the conceptual design through information interaction between the current space, constructed space, base space, and desired space [18]. Liu et al. proposed an integrated process model for product concept design by integrating TRIZ, constraint theory, unforeseen discovery theory, and analogical design to improve the innovation capability of product design [19]. Zheng et al. analyzed the performance characteristics of the product conceptual design process and proposed a model of the conceptual design solution process based on performance evolution [20]. Maher et al. proposed a conceptual design process based on a genetic algorithm, which can model the characteristics of exploratory design and realize the search for problem definition and solution [21]. Wang et al. studied the extension design mode of the rapid configuration of large-scale complex product conceptual design for the multi-level, multi-attribute, and creative product structure configuration process, and proposed a rapid configuration design model for large-scale complex product conceptual design [22]. Kong et al. proposed an integrated life-cycle model for product eco-design in the conceptual design phase, which provides a structured way to represent and manage life-cycle design information and construct a design space covering all feasible design options to provide support for design solution decision making and optimization [23].

Current studies on conceptual design process models are centered on normative studies. In general, the above studies are based on partial stages of the design process and pay insufficient attention to the interactions of workflow, information flow, knowledge flow, and other factors in the conceptual design process model in the actual design process, making it difficult to accurately express the designer’s thinking process in the actual design.

**(2)**  **Research on design iteration and evolution model**
  1) Function–behavior–structure model. To establish a knowledge link between function in the subjective world and structure in the physical world, Gero et al. proposed the function–behavior–structure (FBS) model by separating behavior from function as an intermediate variable between function and structure [24]. Designers establish relationships between function, behavior, and structure of design objects through knowledge experience, attribute function to behavior, and derive behavior from structure, viewing these relationships as different states of the design process. To make the model more consistent with the results of the empirical study, Gero et al. proposed a situated FBS model [3], which extended the original mapping relationship. Based on this model, related researchers have developed the FBS Path model [25], ESBF model [26], FSMEE model [27], FPBS model [28], RFBS model [29], etc. For example, Liu et al. proposed a function–structure concept network construction and analysis method for a smart product design system in order to advance data-driven design, which combines sentence parsing and word/phrase extraction to integrate functional and structural information [30]. The situated FBS model provides the basis for the development of intelligent agent-based design systems and also provides a new perspective for the study of design cognition. However, the mapping provided by the FBS model in dealing with the reconstruction task is only a modification of the structure, desired behavior, and functionality, and does not elaborate on the refactoring of the problem itself and how it is performed.
  2) Co-evolution of problem–solution. To better understand the occurrence mode of creativity, Dorst and Cross conducted a study of design creativity and proposed a theory of co-evolution of problem–solution [31,32]. The theory holds that the design problem solving process is not about solving the problem first and then finding a satisfactory solution, but rather focuses on the ongoing development and refinement of problem constructs and idea constructs, and the ongoing iteration of the analysis, synthesis, and evaluation process between problem and solution. Mao et al. proposed a contradiction solving method for complex product conceptual design based on deep learning and technological evolution patterns, and provided a detailed technology evolution path [33]. In addition, a fully connected DNN model was developed to search for the technological evolution patterns of the expected products, and an evolutionary tree was constructed to generate a final solution to the domain problem based on the predicted patterns. Solution generation is the result of the co-evolution of the problem space and the solution space, and the designer’s goal should be to create “problem–solution” matching pairs. Research by Dorst et al. reveals that innovation solving is an exploratory process, pointing out that the construction of a problem may be more important than the search for a solution. However, although the theory provides the basic cognitive activity of innovation problem solving and the mechanism of innovation solution evolution, it lacks the role of design knowledge in solution evolution and lacks a solution method with operability.
  3) Concept–knowledge theory. Concept–knowledge theory (C-K) considers the design thinking process as a process in which concepts and expertise are interrelated and transformed into each other, developing from initial concepts to precise and accurate expertise and gradually producing feasible solutions [34,35]. C-K theory holds that design tasks aim to transform design propositions that have no logical state in the conceptual space into true propositions that are verified in the knowledge space. In the design process, what drives the joint expansion of the conceptual and knowledge spaces are the four basic operators: C→K, K→C, C→C, and K→K. C–K theory reveals the flow of knowledge in the design process and the way knowledge acts on design concepts and serves as a thinking model to describe the design dynamic mapping process and the generation of new design concepts. However, the theory only explains the operation of thinking and does not give a detailed operational process on how to expand the conceptual space. In addition, although the theory provides a basic way of design process knowledge flow, it lacks a solution method with operability and a specific description of the innovation solution process. In view of this, Li et al. proposed a data-driven reversible framework for the sustainable use of high-value and context-dependent information/knowledge in the development of sustainable smart product service systems [36]. Four steps in this framework, including requirements elicitation, solution recommendation, solution evaluation, and knowledge evolvement, are further introduced to support decision making and optimization in an extended or cyclic life cycle. To solve the knowledge acquisition problem in the product design process, Zhong et al. proposed a requirement-oriented knowledge management framework based on Kansei engineering and knowledge map, and established a demand-oriented knowledge management model using the advantages of Kansei engineering in knowledge acquisition and multi-objective decision making in knowledge selection [37]. These studies have been effective in generating product design solutions, but the absence of an iterative design process makes it difficult to support multiple iterations of requirements–design objectives–solution.
**(3)**  **A brief summary**

Although conceptual design models and evolution models have advanced recently, determining the mapping relationships between design problems, design strategies, and design knowledge during the evolutionary process to accommodate precise knowledge services remains challenging. Several factors have hindered its development and adoption:

  1) Existing studies usually consider the design process as the process of problem solving, focusing more on the generation and evaluation of the problem solving solution itself, and less on the interaction and iterative process between the design problem and the design solution, which is not conducive to the retrospection of the design process and the evolution of the design solution.
  2) In addition to emphasizing the importance of design experience in the generation of design solutions, the role of design strategy and design knowledge in supporting the design process should also be considered.
  3) Design knowledge has different characteristics at different stages of program evolution. It is necessary to study the manifestation of knowledge in each design stage in stages. In addition, each stage of the design process requires corresponding design strategies to provide methodological guidance and corresponding knowledge data to provide information support.

Therefore, Gero et al. proposed a situated Function–Behavior–Structure (sFBS) model of co-design based on the FBS theory that describes the representation of overall co-design activities while retaining a fine-grained representation of each designer’s interaction with their co-designers and their internal cognitive processes [38]. This research team proposed a triple-helix structured model based on problem–knowledge–solution co-evolution for innovative product design process [39], which provides designers with unique innovation design strategies and methods. However, the triple-helix structured model and sFBS theory consider less the role of design strategies in linking design problems and design knowledge.

Based on the existing research, this paper proposes a problem–strategy–solution (PSS) interactive iterative knowledge service method for product design solution evolution based on design strategy as a link, and establishes a PSS-based product design solution evolution by constructing a mapping relationship between design problem, design strategy, and design solution. By constructing a mapping relationship between design problems, design strategies, and design solutions, the PSS-based product design solution evolution knowledge service model is established to assist designers in flexibly applying multi-domain knowledge to achieve the reconstruction and evolution of design solutions. Finally, the design of the magnetic melt spinning device and the fabrication of the strain sensor were completed using the design iteration-based solution evolution method.

## 3. Solution Evolution Process Based on Design Iteration

**Figure 1:**
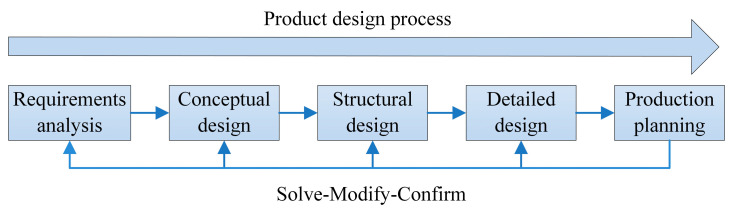
Product design process.

The product design process can usually be divided into five stages: requirements analysis, conceptual design, structural design, detailed design, and production planning [40], as shown in Figure 1.

The requirements analysis phase requires understanding market information and the specific needs of users, i.e., obtaining market resources and determining competitive strategies. Conceptual design is a series of ideas to realize the requirements. This phase is mainly devoted to the main functions of the product, using specific methods to solve the functions that meet specific needs and form a series of design solutions until all the design requirements are realized. Structural design is used to analyze the technical feasibility of the solution generated in the conceptual design phase, so as to carry out the configuration of the main components of the product structure. Detailed design is the design of details for a specific solution. Production planning is to turn the design results of the previous phases into technical documents for production to guide the next manufacturing step.

**Figure 2:**
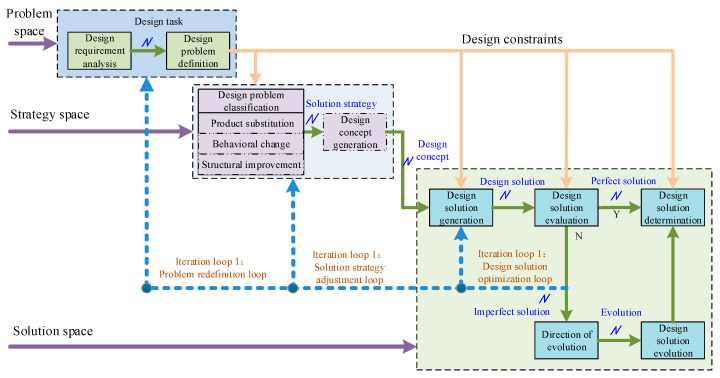
Solution evolution process model based on design iteration.

The product design process is not a simple serialization of these phases, there are intersections and iterations. Product design is a continuous iterative process from problem identification to solution generation, testing, modification, optimization, and determination. The designer usually proposes a solution concept for the design problem, and then uses the solution concept to further understand the design problem and explore a reasonable design solution, i.e., the problem and the solution in the design process are complementary and evolve synergistically [41]. In order to assist designers to clarify the mechanism of scheme evolution and realize product design solution evolution, a solution evolution process model based on design iteration is established in this paper, as shown in Figure 2. With the intervention of problem-solving strategies, design ideas change back and forth between design problems and design solutions, which promotes the simultaneous and interactive evolution of the understanding of design problems and the development of design solutions.

The model is composed of problem space, strategy space, and solution space, and forms three iterative loops for design solution optimization, solution strategy adjustment, and problem redefinition.

(1) Problem space

Design problem space construction mainly includes design requirement analysis and design problem definition. According to different design requirements, product design tasks are transformed into operational design problems, which are the process of problem space construction. The definition of the design problem includes the statement and representation of the problem, i.e., the understanding of the design objectives and tasks, initial conditions and components, and the exploration of the design requirements under the design constraints. By constructing a problem space, it is possible to identify design goals, define design requirements, problems, and constraints, and at the same time limit the range of acceptable design solutions, i.e., the solution space. The problem space is constructed to define the design problem while leaving some free space for the designer to find a suitable design solution through various problem-solving strategies. Therefore, the design problem should not be defined so narrowly as to exclude otherwise feasible problem-solving solutions and compress the solution space. In the product design process, as different types of design solutions are generated, along with their analysis and evaluation, the initial problem definition may then change, i.e., the problem-solving process will in turn affect the problem definition.

(2) Strategy space

The construction of strategy space includes two behaviors: identifying design problem types and generating design concepts. According to the definition of the design problem, first, identify the type of the problem, and further find the corresponding problem-solving method. To assist designers in solving problems in a targeted manner, design problems have been classified into product substitution, behavioral change, and structural improvement according to the change degree of product performance [41]. Product substitution, i.e., changing the type of current product by developing a device that is different from the current product, and which has the functions of the current product and can replace the current product, such as smartphones, or MP3 instead of Walkman. Behavior change does not change the type of the current product, rather it changes the way or principle of the current product function, to achieve the current product function in a different behavior, such as an ultrasonic washing machine using ultrasonic vibration instead of mechanical water stirring. Structural improvements only need to improve the current product by improving a certain component or choosing a certain component to achieve the current product behavior and principle, and no changes to the current product working principle, such as rolling bearing rolling body, have spherical, cylindrical, and other changes in shape. The order of the degree of change in product performance is product substitution > behavioral change > structural improvement. The greater the degree of change in product performance, the greater the design freedom and the wider the scope for solutions, even whimsical ones. The lower the degree of change in product performance, the less design freedom there is, and some otherwise more appropriate solutions may be excluded from the solution space. Using different design strategies for different types of design problems helps designers use the right mindset for product design, thus saving design resources and improving design efficiency.

(3) Solution space

Solution space construction includes solution generation, evaluation, and determination. Under control of the design problem and design constraints, the designer uses corresponding design strategies to develop the generated design concept into a preliminary design solution, evaluates the formed initial design solution according to the design requirements and design tasks, and then further refines and confirms the design solution according to the evaluation results. This process is accompanied by feedback on the design solution, backtracking, and iteration of the design process. The generation and evaluation of design solutions help designers to further understand design problems and adjust design strategies. It can be said that the determination of a design solution is not only the combination of the design concept and the summary of the design solution, but also the evolution of the design solution, which is the result of the interaction of problem space, strategy space, and solution space. That is, the problem and the solution to the problem evolve in concert during the design process.

The problem space, strategy space, and solution space are parallel to each other, and the proceeding and iteration of design activities reflect the state changes of the three spaces, which can be divided into four macro processes.

Process 1: Design Requirements → Design Problem/Design Solution Process. Due to the variability of design tasks, designers analyze design requirements to obtain information about design problems or design solutions.

Process 2: Strategy space → Problem space → Solution space process. (1) The designer constructs the problem space for the design task with the assistance of his or her own design experience and design strategies to define and characterize the design problem initially. (2) The design problem is identified using relevant knowledge to highlight the key and innovation opportunities of the problem from an appropriate perspective. (3) The designer uses the design strategies to solve the problem and obtain a temporary solution, evolving to Process 3.

Process 3: Strategy space → Solution space → Problem space process. (1) With the strategy experience and design knowledge, the provisional solution input to Process 2 is evaluated. (2) If the logical state of the provisional solution is unclear, the design solution is improved, the design strategy is adjusted, or the design problem is redefined using relevant knowledge, and evolves to Process 2. (3) If the logical state of the provisional solution is clear, the output is solution or discarded, and evolves to Process 4.

Process 4: Design concept → strategy space process. The formed design concept can be used as a new strategy to extend the design strategy space.

Among them, Process 2 and Process 3 are circular iterative processes. These four processes demonstrate the iterative evolution between problem space, strategy space, and solution space, which constitute complete innovation-solving activities. These processes are macroscopic, and the mapping process between problem, strategy, and solution at the microscopic level is more representative of the design concept process.

Since product design is an iterative and progressive improvement process, the problem and solution are reconfigured and evolved through multiple loops. In this paper, we focus on three iterative loops: problem redefinition, solution strategy adjustment, and design solution optimization.

(1) Problem redefinition loop

The loop is defined through three spaces: design problem, design strategy, and design solution. When the generated design solution is not good enough, the designer can go back to the problem space to rethink whether the definition of the problem is reasonable. If the original problem is not properly defined, the designer will redefine the problem. In this case, the original problem may be further refined or the problem type may be completely changed; for example, the original structural improvement problem is redefined as a behavior change problem. Design constraints and design strategies change as problem definitions change. The problem redefinition loop changes the strategy space and expands the solution space, and promotes the co-evolution of the problem space, strategy space, and program space. This iterative loop provides designers with greater design freedom and facilitates the generation of innovative solutions.

(2) Solution strategy adjustment loop

This loop goes through the two spaces of design strategy and design solution. If the problem definition is correct, but the existing design solution generated still cannot satisfy the design requirements, the deviation between the two needs to be determined to identify the conflict between the design solution and the design requirements, prompting the designer to re-choose the problem-solving strategy and generate a reasonable design solution. The functionality that can be achieved by existing design solutions can help inspire designers to find multiple behavioral approaches or structural components that achieve the same functionality, and the working principles used by existing design solutions can help designers to find design solutions for related products in other fields. The intertwining of strategy space and solution space contributes to the refinement of the strategy space and can further expand the solution space.

(3) Design solution optimization loop

This loop exists mainly in the solution space, shuttling between the generation and evaluation of design solutions. Design solution optimization mainly means that the designer can directly reuse previously generated good solutions (especially those from other related fields) in solving the problem, or reuse them with minor adjustments. The design solution optimization loop is used to incorporate more existing solutions into the solution space to assist designers in reusing design solutions. New applications of existing solutions in different fields under new design conditions are more helpful for designers to improve design efficiency.

In addition, there are local loops in the product design process, such as the relationship between design problem definition and design requirement analysis, design concept generation, and design problem definition, as well as the relationship between design constraints and each link of the whole design process. The solution evolution iteration loop is a monitoring of the product design process. By continuously defining, evaluating, and reconstructing design problems and design solutions, the designer is prompted to continuously review the design process to ensure the achievement of design goals.

## 4. Evolutionary Mechanism of Product Design Solution Based on PSS Iteration

The designer is the key factor in the innovative design process, converting existing knowledge into design information for artifacts that meet design requirements. The iterations between problem space and solution space in Process 2 and Process 3 are performed with the aid of design strategies. The designer’s knowledge transfer, combination, reasoning, and creation under the action of design thinking are the internal dynamic mechanisms of the interactive evolution of problem–strategy–solution. The idea generation process in innovative design can be understood as the designer is first stimulated by external design information, then reviews and retrieves past design experience and knowledge, adjusts the design process, synthesizes various types of information in the brain, and finally generates a design concept. Experience and outside knowledge influence the designer’s transformation of situations and the processing of information in the creative solution process. It can be seen that the designer’s application and processing of knowledge drive the evolution of problems and solutions.

Therefore, the evolution mechanism of the PSS model is a synergistic problem–strategy–solution evolution: at the initial stage of design, the problem is unclear, abstract, and missing constraints; the corresponding solution is also conceptual and multi-selective; and the knowledge used is principled knowledge. Through interaction, crossover, overlap, and integration, from problem to solution, feedback from solution to problem, and knowledge migration, combination, reasoning, and creation, the problem becomes more and more concrete, and the solution goes from conceptual to comprehensive solution, and the knowledge background goes from general effects and principles to domain and case knowledge, and finally, the problem is clear and structured, forming a perfect solution and generating new knowledge streams and cases. Through the mutual iteration of cognitive activities contained in each of the three spaces, the spiral co-progression of problem reconstruction, knowledge creation, and solution solving is thus realized.

Design iteration-based product design solution evolution includes three mapping relationships: “design problem–solution strategy” mapping, “solution strategy–design solution” mapping, and “design problem–design solution” mapping. These three mapping relationships can be combined into two different mapping processes: the “design problem–design solution” mapping process and the “design problem–solution strategy–design solution” mapping process. In some product design processes, a solution to a problem can be obtained directly from a design problem; i.e., there is a corresponding “design problem–design solution” mapping. Typically, this kind of product is relatively simple, and designers often complete design tasks directly according to the cases mapped from “design problem–design solution”, which exists in the solution optimization iteration loop and is mostly used for design reuse. In the case of complex or innovative product design, it is hard to find a direct “design problem–design solution” mapping, through redefining the problem or analyzing the solution strategy related to the problem, the designer can expand to design thinking. In turn, the problem-to-function analysis and function-to-principle linkage are performed to generate the corresponding design solution. Such a design needs to complete the process from problem to strategy and then to the solution, which exists in the problem redefinition or solution adjustment iteration loop, and is mostly used in the reconstruction and evolution of product design solutions.

To demonstrate the interactive iterative process among the design problem (DP), solution strategy (SS), and design solution (DS), the two representations of an objective state and an expected state to represent them in this paper are defined as follows.

DPo denotes the objectively adopted presentation of the problem definition at this stage.

DPe denotes the expected problem presentation after redefinition.

SSo denotes the solution strategy adopted in the design case or objectively adopted at this stage.

SSe denotes the expected solution strategy after strategy adjustment.

DSo denotes the solution adopted in the design case or objectively adopted at this stage.

DSe denotes the expected design solution after evolution.

**Figure 3:**
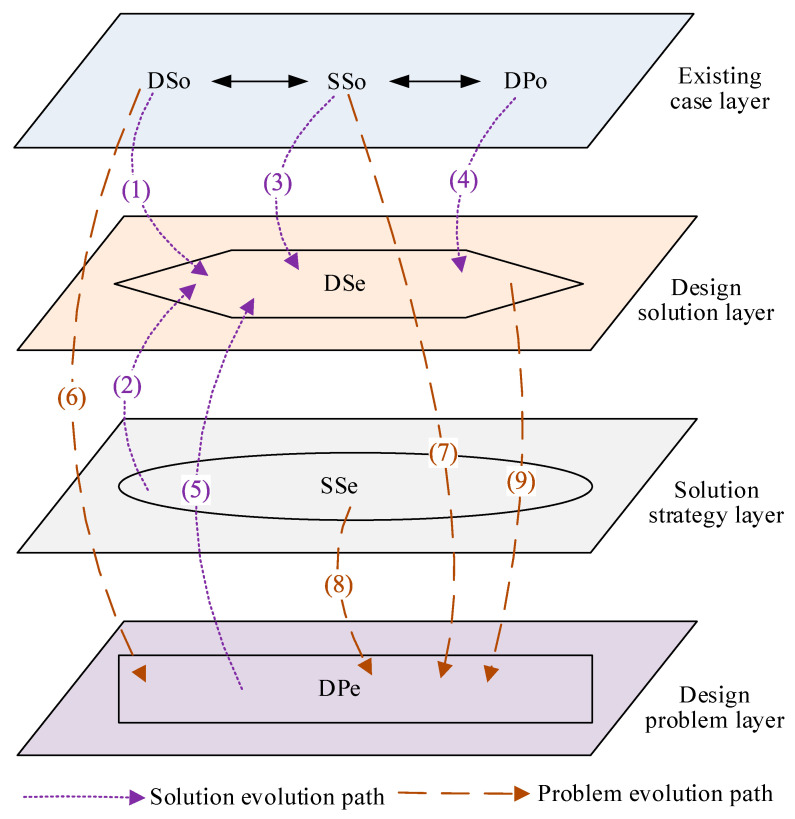
Evolution process of PSS-based product design solution.

The product design solution evolution process based on problem–strategy–solution (PSS) iterations constructed based on the mapping relationship among design problem, solution strategy, and design solution is shown in Figure 3, and the evolution paths of the three together form the design iteration and evolution process.

Solution evolution path:

(1) DSo→DSe: A new design solution can be obtained from the identified design solution by structural improvement or parameter adjustment, etc.
(2) SSe→DSe: A new design solution can be obtained by mapping the expected solution strategy to the design solution library.
(3) SSo→DSe: A new design solution can be obtained by mapping the solution strategy adopted in the identified design case to the design solution library.
(4) DPo→DSe: A new design solution can be obtained by directly mapping the design solution library from the defined problem definition, and this method is suitable for experienced designers.
(5) DPe→DSe: A new design solution can be obtained by directly mapping the design solution library from the expected problem definition, and this method is suitable for simple problem solving or product repetition design.

Problem evolution path:

(6) DSo→DPe: Mapping from the identified design case to the design problem library to achieve the redefinition of the design problem.
(7) SSo→DPe: Mapping from the adopted solution strategy in the identified design case to the design problem library to achieve the redefinition of the design problem.
(8) SSe→DPe: Mapping from the expected solution strategy to the design problem library to achieve the redefinition of the design problem.
(9) DSe→DPe: Mapping from the expected design solution to the design problem library to realize the redefinition of the design problem.

In the above mapping process, the solution strategy is the bridging link from the problem space to the solution space, and the design knowledge is the basic resource to realize the problem evolution and solution evolution. With the role of strategy service and knowledge service, the interaction of problems and solutions is driven forward through the migration, combination, and analogy of knowledge. In a specific product design process, the frequency of the above-mentioned evolutionary paths is mainly determined by the complexity and innovativeness of the product, the more complex and innovative the product is, the more iterations and mappings are required.

Therefore, the evolution mechanism of the PSS model is a synergistic problem–strategy–solution evolution. In the initial stage of design, the problem is unclear, abstract, and missing constraints, and the corresponding solution is conceptual and multi-selective, using design strategies that mainly reuse existing case knowledge. Through the iteration of the problem and solution and the migration and combination of design knowledge, the design problem becomes more and more concrete, the design solution also changes from conceptual ideas to comprehensive solutions, and the design strategy also changes from reusing design cases to using scientific effects and inventive principles, and finally, the design problem becomes clear and structured, forming a perfect solution and generating new knowledge and cases. Through the mutual iteration of the three spaces, the spiral co-development of problem reconstruction, knowledge creation, and solution solving is realized.

## 5. Product Design Solution Evolution Knowledge Service Model

### 5.1. Product Design Solution Evolution Knowledge Service Dimension Construction

Many design problems will be encountered in the process of product design solution evolution. When a designer encounters a problem, he or she expects to easily arrive at a solution to the problem without understanding the problem. Problems that have been “solved” with hasty “emergency” solutions are bound to be changed again later. In order to assist designers to better solve the problem and achieve the reconstruction and evolution of the solution, it is necessary to conduct an in-depth analysis of the problems of the evolution of the product design solution.

**Figure 4:**
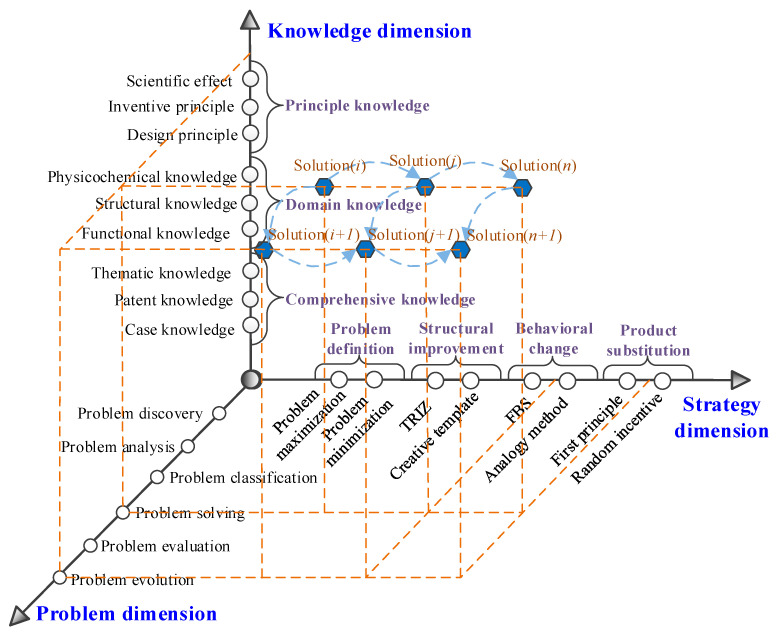
Product design solution evolution knowledge service dimension.

In the process of problem–solution co-evolution, the discovery, analysis, and definition of product design problems, the selection of design strategies, the solution of design problems, and the generation of design solutions all require knowledge support. Three conditions are necessary to realize the knowledge service for product design solution evolution [42]: (1) a complete problem-solving process; (2) appropriate design strategies and design methods to guide the designer’s creative thinking; and (3) appropriate knowledge resources to assist the designer in invoking the knowledge. Therefore, this paper constructs a product design solution evolution knowledge service framework in three dimensions: problem dimension, strategy dimension, and knowledge dimension, as shown in Figure 4.

(1) Problem dimension

The problem dimension covers the whole process of problem discovery, problem analysis, problem classification, problem solving, problem evaluation, problem evolution, and other series of activities in the product design process, and it is the micro-history of product design solution generation and development. In the phases of problem discovery, problem solving, and problem evolution, the research objectives and objects are different. Solution evolution knowledge service is to achieve design solution evolution and complete product design tasks by interacting and mapping this dimension with the other two dimensions and feeding the knowledge and strategies of the other two dimensions into various stages of design problem discovery, analysis, and solution to assist designers in design decision making.

(2) Strategy dimension

The strategy dimension, i.e., the methods and strategies required to solve the design problem, provides the corresponding methodological support for the generation and evolution of product design solutions. After obtaining the design requirements or the design problem to be solved in the design task, we should first decide how to understand the design problem, i.e., the problem definition. In this paper, two strategies, problem maximization and problem minimization, are used to assist the designer in defining the problem. Problem maximization refers to where the designer expands the scope of the problem by asking, “what is the purpose of solving the problem” and “is there any other way to achieve the same purpose”, etc. Problem minimization means that the designer focuses on a specific problem by asking, “what are the obstacles to solve this problem” and “how to remove this obstacle”. Due to the polymorphic nature of design problems (structural improvement problems, behavior change problems, and product substitution problems), targeted design strategies need to be selected based on problem types, such as TRIZ theory and the creative template method for solving structural improvement problems, the FBS theory and analogy method for solving behavior change problems, and first principle and random incentive for solving product substitution problems to improve the efficiency of problem solving.

(3) Knowledge dimension

The knowledge dimension is all the knowledge resources required in the process of product design problem solving. Product design knowledge is characterized by various forms and complex structures, and the types of knowledge required are different in different design stages. In order to extract the knowledge required for design from the massive design resources, a clear classification (principle knowledge, domain knowledge, and comprehensive knowledge) and organization and management of knowledge are needed to realize the query and retrieval of knowledge and the active push of knowledge by designers during the evolution of design solutions [43], and to help designers acquire and flexibly apply multi-domain design knowledge for design decisions. The principle knowledge includes design principles, scientific effects, inventive principles, etc., which are used to assist designers in forming evolutionary strategies; domain knowledge includes functional knowledge, structural knowledge, physicochemical knowledge, etc., which are used to assist designers in acquiring the basic principles for realizing the behavioral state of products; and comprehensive knowledge includes design cases, patent knowledge, thematic knowledge, etc., which provide designers with design reference and design verification, etc.

In the process of forming and gradually expanding the design problem space and the design solution space, the design strategy space is the link between the two. With the support of knowledge, through the interaction, intersection, and integration of problem space, strategy space, and solution space, from design solution feedback to strategy and then to problem, the problem definition becomes clearer and clearer, the strategy becomes more and more targeted, the design solution becomes more and more comprehensive, and, finally, the problem definition becomes clearer and clearer, new design strategies are mined, perfect design solutions are formed, and even new knowledge is generated. The design problem, design strategy, and design case spiral together and support each other to complete the stimulation of the product design solution evolution process.

### 5.2. Knowledge Service Model for Solution Evolution Based on Design Iteration

**Figure 5:**
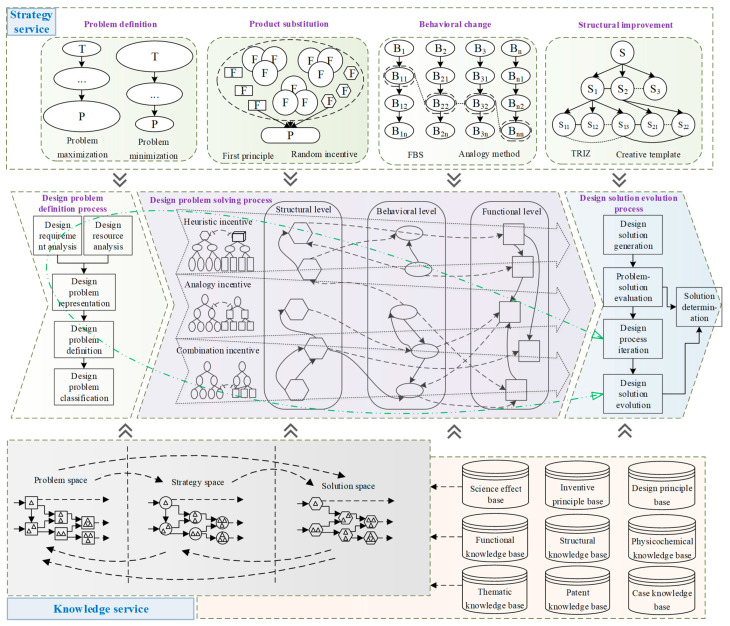
Knowledge service model of solution evolution based on design iteration.

Given that knowledge has different characteristics at different stages of solution evolution, it is studied in stages to examine how knowledge is expressed at each design stage and how it can be efficiently applied. Based on the design iteration process shown in Figure 2, a product design solution evolution knowledge service model is established to study the knowledge service model in three stages: problem definition, problem solving, and solution evolution. Each stage requires appropriate design strategies to provide methodological guidance and knowledge to provide information support. Each stage of the design solution evolution process requires policy services and knowledge services to establish mapping relationships to support the evolution process with services, as shown in Figure 5.

(1) Problem definition stage

Defining the problem correctly is a prerequisite for efficient problem solving. Analysis of the design task from the perspective of design requirements and design resources helps to identify the key problems of product design and prepare for the next step of problem solving. The specific process is as follows: (i) Collecting design requirements, design resources, and design constraints as comprehensively as possible, clarifying design tasks and existing design conditions, and obtaining the initial state of the design problem. (ii) With the support of design problem analysis strategy services, such as problem expansion and problem reduction, the design problem is characterized and problem definition is completed. (iii) Classifying the design problem into product substitution problem, behavior change problem, and structure improvement problem according to the definition of the design problem.

(2) Problem-solving stage

Product design problems involve multiple aspects such as existing product structure improvement, product behavior change, product substitution, etc. Different problems require appropriate design strategies to provide methodological guidance. Combined with the problem types identified in the previous stage, the combination of incentives solving, analogy incentives solving, and heuristic incentives solving strategies are used to stimulate designers’ innovative thinking to solve design problems from the perspective of product function, behavior, and structure.

Combination incentive: The combination incentive strategy is suitable for the improvement and reorganization of the product structure. Such problems often arise because designers lack the necessary knowledge of innovative methods. The combined incentive method provides designers with a multi-directional strategy for solving product structure problems, classifies and solves typical product structure problems, and provides the corresponding design knowledge as support. The designer can choose the appropriate service strategy and knowledge according to the problem identification. The combined incentive strategy mainly includes TRIZ service strategy, Creativity Templates (CT) service strategy, etc. This strategy can assist designers to call functional knowledge, structural knowledge, scientific effect knowledge, inventive principle knowledge, etc. When there is a technical conflict or physical conflict in a product, TRIZ service strategy can map out the corresponding knowledge of inventive principle and technical evolution theory to assist designers in resolving the conflict. When there is a need to improve the function of the product, the creative template service strategy can map out the corresponding structural knowledge to assist the designer in improving the function.

Analogy incentive: Analogy incentive strategy is applicable to the scenario where the existing working principle and behavior mode need to be improved in order to realize a specific function of the product. Analogy incentive strategy starts from the product function, analyzes the working principle and behavior of existing products, and uses the analogical thinking method to apply design cases from other fields or multidisciplinary knowledge to the product design process, and generates innovative solutions after combination and comparison. Analogy incentive strategies mainly include Function–Behavior–Structure (FBS) service strategies, analogical service strategies, etc., which guide designers in knowledge migration. When a new working principle needs to be established to realize product functions, the FBS service strategy can map out the corresponding functional knowledge, structural knowledge, scientific effects, and physicochemical knowledge to assist designers in realizing product functions. The analogy service strategy can map out multi-domain knowledge to provide designers with case references and guide them to think by analogy.

Heuristic incentive: Heuristic incentive strategy is applicable to design problems without a specific goal, where the purpose of such designs is mainly to replace the current product, but there is no clear concept of a new product in the future. The implementation of the heuristic incentive strategy mainly stems from the external stimulation of the designer by the knowledge information. When faced with such design problems, the heuristic incentive strategy will push a large amount of knowledge (including images, text, videos, animations, etc.) related or unrelated to the design problem to the designer, and this knowledge combined with the design task motivates the designer to make forced associations, thus inducing the designer to generate ideas. The main ones suitable for heuristic incentive strategies are first-principle strategies, random incentive strategies, etc. The first-principle strategy guides designers to trace the essence of design and to clarify product functions. The random incentive service strategy can map out a large number of knowledge fragments to force designers to make associations and inspire them to generate innovative solutions.

(3) Solution evolution stage

After the problem-solving stage, the designer generates several design solutions that need to be evaluated, reconfigured, and evolved. Based on the evaluation of the generated design solutions, the matching and deviation between the design problem space and the design solution space are analyzed to determine whether the current design solution leads to new design problems or contradictions and conflicts. At the same time, the design problem space and the design solution space continuously collide and interact to help designers discover previously undiscovered design problems and design constraints. After solution evaluation, the design solution that meets the design requirements is identified as the final solution for design solution output. If the design solution cannot meet the design requirements or completely solve the design problem, then, based on the three iterative loops of problem redefinition, solution strategy adjustment, and design solution optimization, or return to the starting state of the design to redefine the design problem and carry out a new round of problem solving, or change the problem solving strategy, or re-select the design solution, the solution will be designed back and improved repeatedly to achieve the reconstruction and evolution of the design solution.

### 5.3. Knowledge Matching

Due to the complexity of product design knowledge itself and the diversity of knowledge required in different design stages, in order to realize knowledge services for the evolution process of design solutions, it is necessary to build a knowledge base and form a unified knowledge management model to support and record the knowledge application and the iteration and evolution process of design solutions. According to the hierarchical division in the problem–solution interaction-driven product design solution mapping process, we construct a solution strategy repository and design knowledge repository, respectively, and support the reconstruction and evolution of product design solutions through strategy service and knowledge service together.

The policy service can be expressed as

*SSs = (SA SR SP SM SK SI)* (1)

where *SA* represents the concept of solution strategy, including the name, number, storage state, and description of the solution strategy in natural language; *SR* represents the logical relationship between solution strategies, which is a collection of strategy-to-strategy mappings, indicating that multiple design strategies work together on the same design problem, and is used to provide a reference for designers when choosing a solution strategy; *SP* denotes the set of problems that the solution strategy can solve, and indicates how well the solution strategy matches with the product substitution problem, the behavior change problem, and the structure improvement problem; *SM* denotes the access and execution method of this solution strategy; *SK* represents the ensemble of mapping relations between this solution strategy execution and the required knowledge services; *SI* represents the set of cases related to this solution, and the expressions of these cases are also normalized to contain the case name, the case description, the design goal of the case, the product requirements of the case, the problem description of the case, the functional features in the case, the configuration and structure of the parts in the case, the design strategy of the case, etc.

Knowledge services can be expressed as

*DKs = (KA KO KP KK KS KI)* (2)

where *KA* denotes the initial knowledge resources, such as web pages, pictures, and other knowledge without normative expression, and is the sum of all basic knowledge; *KO* denotes the rules of knowledge normative expression, and the knowledge resources in a uniform format can be obtained after normative expression, taking the principle knowledge as an example, including the principle knowledge label, the principle knowledge description, the set of functions that can be achieved by the principle knowledge, the set of behavioral effects and structures related to the principle knowledge, etc.; *KP* denotes the set of problems that can be solved by the knowledge service, indicating the matching degree between this knowledge and the product substitution problem, behavior change problem, and structure improvement problem; *KK* denotes the set of mapping relations between this knowledge and other knowledge, which is multiple knowledge acting together in the same strategy service; *KS* represents the set of policy service mapping relations associated with this knowledge service, and assists the designer in selecting the policy service through the knowledge service; *KI* denotes the collection of cases related to this knowledge, and the expressions of these cases are also normalized, including the case name, case description, design objective of the case, product requirements of the case, problem description of the case, functional features in the case, part configuration and structure in the case, design strategy of the case, etc.

In this paper, we use the existing knowledge pushing algorithm of the research team for policy service and knowledge service matching operation [44]. The matching idea can be described as follows: the item *PX* to be matched (which can be design problem *DP*, solution strategy SS, or design solution *DS*) is decomposed into multiple subitems *PX_i_*, which are matched and computed with matching items *PY* (which can be strategy service SSs or knowledge service *DKSs*), and the design task is marked as completed when all subitems *PX_i_* are satisfied or solved. The matching function can be expressed as *m_p_(MX_P_, PX_i_, PX_A_)* → *(PX_i_, PY)*, where *m_p_* denotes the mapping function, *MX_P_* denotes the textual representation of the item to be matched, *PX_i_* denotes the subitems to be matched, *PX_A_* denotes the axiom to be followed with the matched item, and *(PX_i_, PY)* denotes the matching result. *m_p_(MX_P_, PX_i_, PX_A_)* → *(PX_i_, PY)* denotes the matching result if the parameters such as *MX_P_*, *PX_i,_* and *PX_A_* into the mapping process shown in Figure 3, the matching result *(PX_i_, PY)* will be output by the mapping from the problem evolution path or solution evolution path according to the actual situation. Then, the designer will carry out problem solving and solution evolution with the assistance of strategy service and knowledge service, and finally complete the design task.

## 6. Case Study

**Figure 6:**
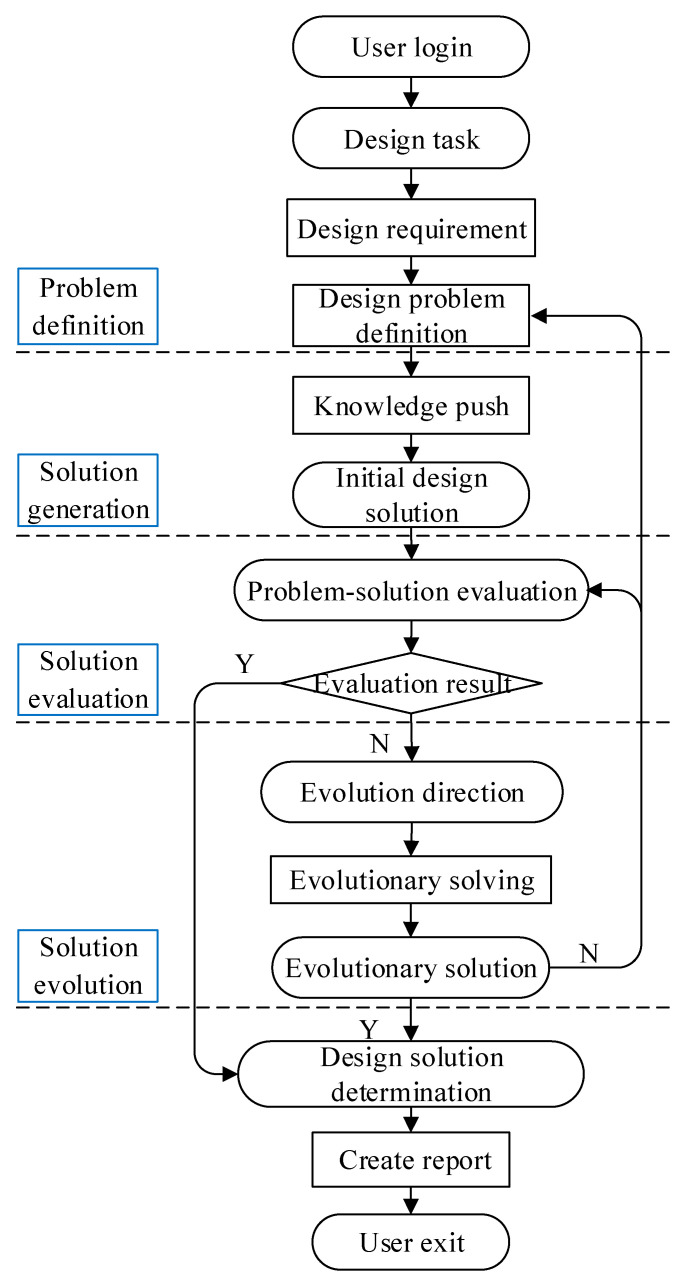
System operation flow.

The product design solution evolution was systematically implemented as a module of the existing computer-aided product innovation design knowledge service system of the group [44], and the operation flow of the system is shown in Figure 6.

(1) Problem definition

After acquiring the product design task, the designer needs to define the design requirements and design problems, and determine the functions that the product must have according to the design requirements and design problems, so as to clarify the design objectives.

(2) Solution generation

According to the product function, design strategy knowledge, domain knowledge, and comprehensive knowledge are pushed to the designer to assist the designer in quickly generating one or more initial design solutions.

(3) Solution evaluation

For the initial design solution, the design problem space and the design solution space are analyzed separately to determine whether the current design solution leads to new design problems or contradictions and conflicts, and to help designers discover design problems that have not been considered before, and to realize the reconstruction of design problems through the interactive drive of design problems and design solutions, so as to determine the evolutionary direction of design solutions.

(4) Solution evolution

The evaluation results are determined. If the design problem is defined incorrectly, the design problem is reconstructed. If the design problem is accurately defined, but the design solution and the design problem still cannot be matched, the deviations existing between them are determined, and a new design solution is generated through the combination and migration of knowledge/principles, thus realizing the evolution of the design solution.

The process of product design solution evolution is not a simple serial of these stages, there are crossovers and iterations, it is a continuous iterative process of “solve–modify–confirm”, accompanied by the reconstruction of the design problem and the generation, retracing, and improvement of the design solution.

To demonstrate the solution evolution approach in this paper, the design process of a magnetic melt spinning device is used as an example. The spinning device is mainly used for the preparation of nanofibers. The preparation of nanofibers involves knowledge in multiple domains such as mechanics, materials, electromagnetism, etc. The amount of knowledge is huge, the speed of knowledge update is fast and specialized, and designers require high efficiency and accuracy of knowledge services. Therefore, the feasibility and effectiveness of the evolutionary knowledge service approach to product design solutions described in this paper can be well verified by applying it to the design process of magnetic melt spinning devices.

**Figure 7:**
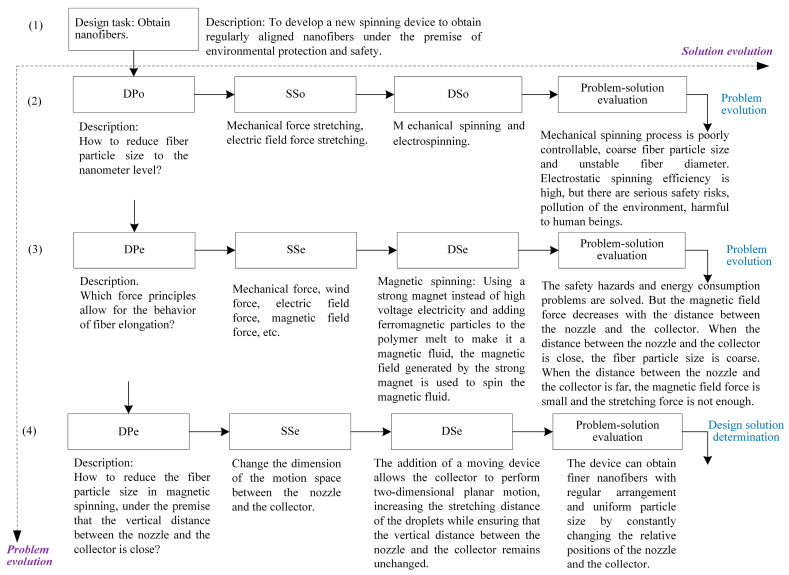
Solution evolution process of the magnetic spinning device.

Nanofibers occupy an irreplaceable position in the fields of composites, sensing, etc. [45]. Electrospinning is currently the most commonly used method for the preparation of nanofibers [46,47]. Electrospinning is a spinning method that uses electrostatic force to produce nanofibers. The key to this method is to make the polymer solution deformation and jet motion in a high-voltage electrostatic field, and the solvent volatilizes during the jet motion to solidify the polymer to obtain nanofibers. However, with the improvement in environmental protection and safety requirements, the electrospinning method still has more and more obvious defects, such as the safety hazards caused by high voltage, a disorder of nanofiber arrangement caused by the bending instability stage, environmental pollution, and harmfulness to the human body caused by organic solvents. Therefore, there is an urgent need to develop a new type of spinning device. As a member of the design team, the authors applied the PSS-based product design solution evolution technique to the design of a new spinning device while participating in a nanofiber preparation project in a laboratory. The specific application process is shown in Figure 7.

(1) Design Task

To develop a new spinning device to obtain regularly arranged nanofibers under the premise of environmental protection and safety.

**Figure 8:**
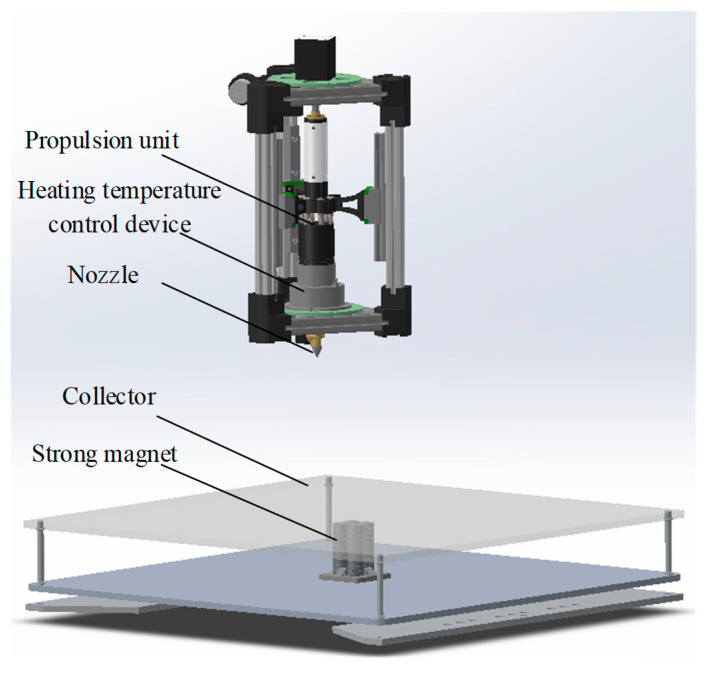
Schematic diagram of magnetic spinning solution.

**Figure 9:**
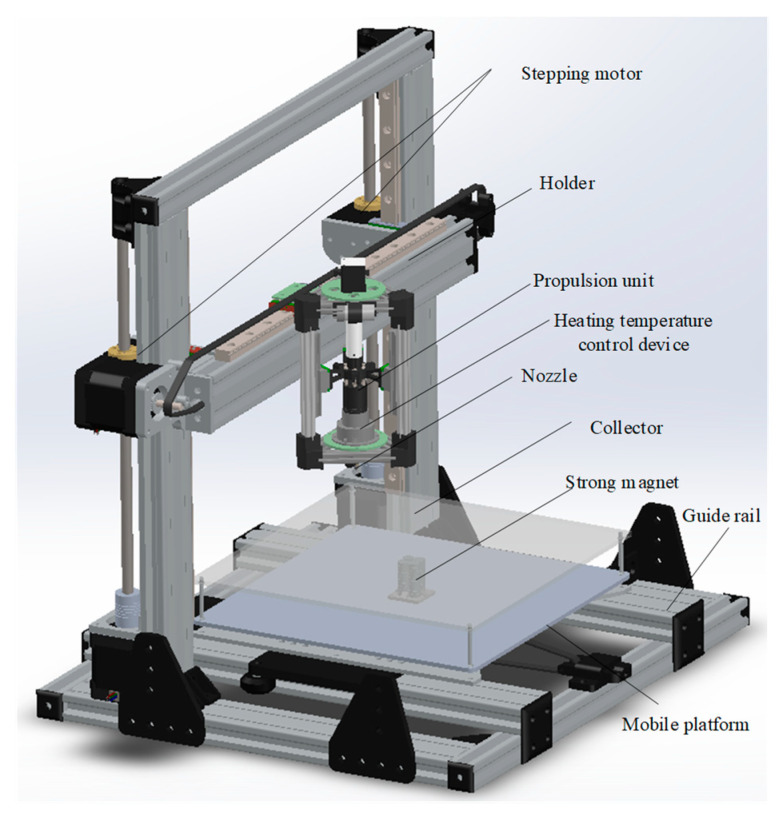
Magnetic spinning device.

(2) Initial solution generation
  - Problem definition: The degree of stretching of fiber particle size directly affects the quality of nanofibers. Thus, the design problem is defined as “How to reduce the fiber particle size to the nanoscale?” based on the above design task.
  - Formation of the initial solution: According to the problem definition, knowledge is pushed from the product innovation design knowledge service system to obtain the design cases that satisfy the conditions. The cases of mechanical spinning and electrospinning are obtained by the product innovation design knowledge service system. The pushed cases provide theoretical guidance and reference for the solution design. According to the pushing result, two initial solutions are obtained: mechanical spinning and electrospinning.
  - Initial solution evaluation: After the analysis of the initial solution, it was found that the traditional mechanical stretch spinning process is poorly controllable; regarding the preparation of coarse fiber particle size, only the preparation of conventional fineness of the fiber is possible, while the obtained fiber diameter is not stable, having more defects and other problems. Although electrospinning has the advantages of simple structure and high production efficiency, there are still some defects: (1) In order to generate sufficient electrostatic force, tens of thousands of volts of a high-voltage electrostatic field are required, which not only greatly increases the cost of industrial production, but there are also serious safety risks. (2) Spinning process will occur in the bending instability stage, which will lead to mutual repulsion of the deposited nanofibers, and it is difficult to obtain regular patterns. (3) The dissolution of the polymer requires organic solvents, which increases environmental pollution and harmfulness to humans.
  - Determination of evolution direction: Through the evaluation and analysis of the initial solution, it is found that although the initial solution can reduce the fiber particle size to the nanometer level, there are still various problems in the practical application, and a new spinning principle is needed.
(3) Problem–solution evolution
  - Problem evolution: According to the problem evolution path (6) shown in Figure 3, the defects of the two initial solutions stem from their use of two different tensile principles of mechanical and electrostatic forces; to solve the various problems with mechanical and electrostatic spinning as the initial solution, it is necessary to change the tensile force principle of spinning. Therefore, the evolved design problem can be defined as “which force principle can achieve the behavior of fiber elongation”. According to the description of the strategy space in Figure 2, this problem is a behavior change problem.
  - Forming an evolution solution: According to the analysis of strategy dimension in Figure 4, the behavior change problem can be solved by using the FBS theory and analogy method. According to the analysis of the solution strategy in Figure 5, the FBS service strategy in the analogy incentive strategy is proposed to be used to push the corresponding functional knowledge, structural knowledge, scientific effect, physicochemical knowledge, etc. The working principle and behavior of the existing spinning device will be changed according to the pushed knowledge. According to the solution evolution path (2) shown in Figure 3, knowledge is pushed in the product innovation design knowledge service system to obtain the principles that can realize the fiber stretching function and mainly include mechanical force, wind force, electric field force, magnetic field force, etc. Based on the analysis of the pushed knowledge principles and above-mentioned, it can be seen that spinning stretching with mechanical force and electric field force as the principle has a variety of problems. At the same time, the analysis found that when applying the wind spinning scheme, in the spinning process, not only is the solvent volatilization fast, but the deposition range is wide and uncontrollable, and the wind direction is difficult to keep stable, which easily leads to dripping material, hanging, and other problems, which seriously affects the continuity of the spinning process. Therefore, after screening, the evolutionary scheme was determined to be magnetic spinning. The preliminary three-dimensional model of magnetic spinning established is shown in Figure 8.
  - Evolution solution evaluation: The magnetic spinning solution solves the problems of safety hazards and low energy consumption in electrostatic spinning. However, the magnetic field force decreases as the distance between the nozzle and the collector increases. When the vertical distance between the nozzle and the collector is close, the fiber stretching process is not sufficient, and the fibers are deposited in the collector before reaching the nanometer level, and the fiber particle size is coarse. When the vertical distance between the nozzle and the collector is far, the magnetic field force is weak, and it is difficult to form sufficient stretching force.
  - Determination of evolution direction: After the evaluation of the magnetic spinning device, it is found that the position of the collector and nozzle is fixed, the distance between the two is large, the magnetic field is weak and cannot provide a large enough stretching force; small distance, the magnetic field is strong, but the stretching distance is not enough, it is difficult to obtain the nano-scale particle size. The problem with the magnetic spinning devices is that the fixed magnetic field force cannot reduce the fiber particle size to the ideal size, and the evolutionary direction of the magnetic spinning solution is particle size reduction.
(4) Second problem–solution evolution
  - Problem evolution: Although the magnetic spinning solution solves the problems of poor safety and irregular shape of patterned fibers in electrospinning, there is also the problem of coarse fiber particle size. According to the problem evolution path (9) shown in Figure 3, the second evolutionary problem was defined as “how to reduce the fiber particle size in magnetic spinning, under the premise that the vertical distance between the nozzle and the collector is close“ for each problem and evolutionary direction found after the evaluation of the first evolutionary solution. According to the description of the strategy space in Figure 2, this problem is a structural improvement problem.
  - Formation of the second evolution solution: According to the analysis of strategy dimension in Figure 4, the structural improvement problem can be solved by the TRIZ theory with the creative template method. According to the analysis of the solution strategy in Figure 5, the TRIZ service strategy in the combination incentive strategy is proposed to be used to push the corresponding knowledge of invention principles and technical evolution theory. The structure of the existing spinning solution can be improved according to the pushed knowledge. According to the solution evolution path (1) shown in Figure 3, the contradictions of the existing magnetic spinning solution are analyzed: the distance between the collector and the nozzle is large, the magnetic field force is weak and cannot provide sufficient stretching force; the distance is small, the magnetic field is strong, but the stretching distance is not enough to obtain the nanoscale particle size. This is a typical set of technical conflicts, which can be solved with the help of the inventive principle in the TRIZ theory [14]. The specific steps are as follows: to ensure that the fiber particle size to nanometer, it must be made subject to the magnetic field force that has a large enough, “force” for the parameters to be improved, corresponding to the general technical parameters “10—force”. However, in order to increase the magnetic field force, the vertical spinning distance between the nozzle and the collector should be small enough, resulting in the deposition of droplets on the collector before they are stretched long enough from the nozzle, and the fiber particle size cannot reach the nanometer level. That is, the “droplet stretching length” becomes a deteriorating parameter, corresponding to the general technical parameter “3—length of the moving object”. After consulting the conflict matrix, we obtained 4 recommended inventive principles: 9 (pre-reaction), 17 (spatial dimensional change), 19 (periodic action), and 36 (phase change). After analysis and comparison, finally, the inventive principle 17 (spatial dimension change) was selected for the problem solution. This problem is solved by a three-dimensional movement between the magnetic fluid injection device and the collector. The addition of a moving device allows the collector to perform a two-dimensional planar motion, increasing the stretching distance of the droplets while ensuring that the vertical distance between the nozzle and the collector remains unchanged. The improved solution changes the original fixed magnetic spinning device into a three-dimensional magnetic spinning device that can move up and down, left and right, and back and forth, as shown in Figure 9.
  - Evaluation of the second evolution solution: The device can obtain finer nanofibers with regular arrangement and uniform particle size by constantly changing the relative positions of the nozzle and the collector.

## 7. Experiment

### 7.1. Magnetic Spinning

**Figure 10:**
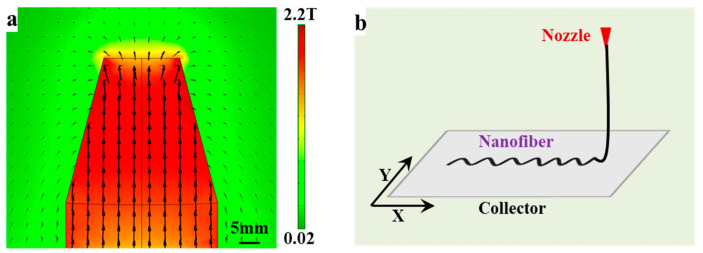
(**a**) Magnetic spinning device. (**b**) Magnetic spinning simulation diagram.

Polystyrene (PS, MW = 200 kDa) was chosen as spinning material; the average size of Fe_3_O_4_ nanoparticles was 50 nm and the blending ratio of Fe_3_O_4_ in PS nanofiber was set as 5%. The nanofibers were prepared through the customized magnetic spinning device, as shown in Figure 9. Comsol simulation was applied to calculate the magnetic field in Figure 10a, which indicated that a magnetic field of 0.02–2.1 T was generated around the surrounding area. The magnetic field exceeded 1.6 T in the range of 5 mm around the nozzle, where magnetic spinning occurred [48].

Two g of PS was dissolved in eight mL of dimethylformamide (DMF), and then stirred for 8 h in water bath at 80 °C to obtain pure PS solution. Then, 0.1 g Fe_3_O_4_ nanoparticles was added to 2 mL DMF, and the mixture was sonicated for 4 h. Then, the pure PS solution was blended with Fe_3_O_4_ nanoparticles dispersion and stirred for another 4 h. Finally, the homogeneous mixed solution was placed in a 40 °C drying oven. After the acetone had completely evaporated, a PS@Fe_3_O_4_ mixture compound was obtained and then loaded into the melt chamber, and the magnetic mixture in the molten state was obtained by heating to 210 °C. The propulsion device extruded the mixture from the nozzle at a speed of 0.4 mL/h to form droplets. The distance between the nozzle and the collector (120 × 120 mm) was set as 5 mm. Simultaneously, the collector was fixed on the platform, which moved according to the set trajectory within 100 mm at a speed of 0–100 mm/s with positional accuracy of 0.01 mm, as shown in Figure 10b [49]. Meanwhile, the motion generates additional mechanical stretching force to further reduce fiber diameter. Fibrous strips with length of 10 mm were prepared through direct-writing curved nanofiber repeatedly.

### 7.2. Construction of Strain Sensor

**Figure 11:**
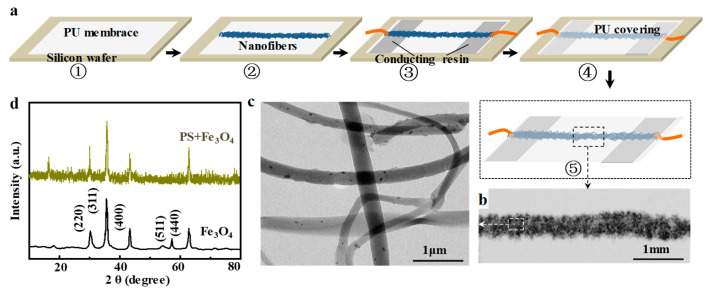
(**a**) Schematic depiction of the fabricating process of strain sensor with nanofiber strips. (**b**) Optical image of fibrous strips from magnetic spinning. (**c**) TEM image of PS@Fe3O4 nanofibers. (**d**) XRD patterns of PS and PS- Fe3O4 composite nanofibers.

Strain sensor was constructed with PS@Fe_3_O_4_ fibrous strips; assembly process is shown in Figure 11a. A PU membrane with thickness of 20 μm was attached to silicon wafer, which was used as nanofiber collector, followed by direct-writing composite nanofiber on it, then two copper wires were pasted to the ends of fibrous strips with conducting resin (5 × 2 mm) and another PU membrane was covered to capsulate the fibrous sensor [50].

The macroscopical morphology of composite nanofibers was observed using a stereo microscope (Zeiss Axio Lab, Weztlar, Germany). As shown in Figure 11b, fibrous strips with average width of 0.5 mm and thickness of 50μm were composed of PS@Fe_3_O_4_ nanofiber with curved morphology, which is beneficial to bear tensile strain. Microtopography of nanofiber was determined by transmission electron microscope (TEM; JEOL JEM-2100F, Japan). Figure 11c shows a typical TEM image of composite nanofiber, indicating around 400 nm in diameter and Fe_3_O_4_ nanoparticle with diameter of 50 nm uniformly dispersed in nanofiber. X-ray diffractometer (XRD, Philips X’Pert PRO, Almelo, The Netherlands) analyses were used to characterize the crystalline profile of nanofiber, as shown in Figure 11d. Distinctive characteristic peaks appear at 2θ of 30.2°, 35.6°, 43.2°, 57°, and 62.6°, corresponding to the five crystal planes (200), (311), (400), (511), and (440) in the Fe_3_O_4_ crystal structure, which proves that the Fe_3_O_4_ nanoparticles are successfully encapsulated in nanofibers [51]. Nanoparticle distribution reduced the resistance of nanofibers, and the electrical conductivity of fibrous strips achieved a certain value to be applied as strain sensor.

### 7.3. Characterization of Strain Sensor

Stretchable electronics have aroused attention for potential applications including, but not limited to, deformable, foldable, rollable, and bendable displays. Stretchable strain sensors are essential parts of these electronics, and attempts have been made to develop different fabrication methods and achieve stable performances. One of the most formidable challenges of the stretchable sensors is the retention of high conductivity even under severe deformation [52]. The application of nonwoven membranes is limited by their disordered fiber positioning. Herein, fibrous strips made from PS@Fe_3_O_4_ blends were investigated as substrates for strain sensor.

**Figure 12:**
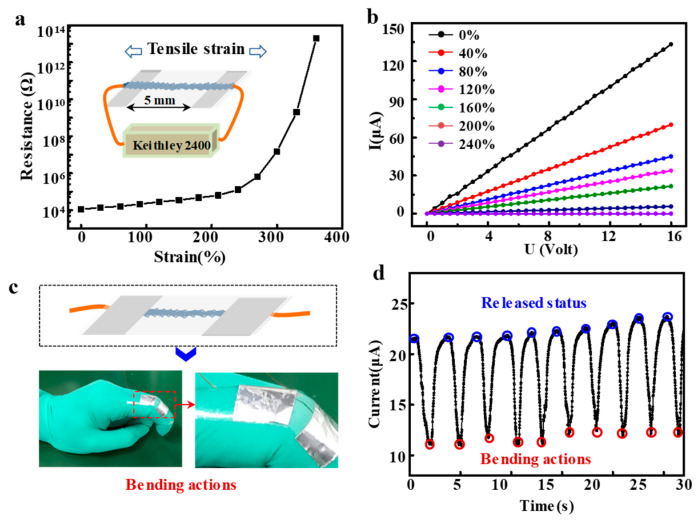
(**a**) Resistances of strain sensors with different tensile strains. (**b**) I–V characteristics of strain sensor under different tensile strains. (**c**) Images of forefingers assembled with fibrous sensors to record the electric current strength during the bending and release actions. (**d**) Electric current strength changes of the fibrous sensors after different cycles of bending and release.

It is well known that the performance of a strain sensor is characterized by a gauge factor for practical application, which is defined as (dR/R)/(dL/L), where R is the resistance of the sensor, L is the distance between the electrodes on the sensor (5 mm), and dR and dL are the resistance and length changes of sensors during stretching, respectively. To measure the sensitivity of the sensor to strains, the fibrous blocks were stretched from 0 to 240% by a customized stretching apparatus along the strips’ direction (Figure 12a). During the initial stage, resistance of the fibrous strips exhibited few changes during the stretching process. As strain increased from 0 to 240%, the resistance value increased slowly, indicating stable electrical conductivity of sensor. The resistance increased sharply when the tensile strain exceeded 240%, which indicated that the conductive integrity was destroyed as too large a distortion occurred within composite fibers.

Figure 12b shows the I–V characteristics of fibrous sensors with different uniaxial strains. The electric current strengths decreased gradually with the strain varying from 0 to 240%, but the resistance of the sensor indicated no change at the same strains. The electrical resistance of highly aligned fiber arrays was only sensitive within limited strains and fluctuations of resistances were observed as strain beyond the limits [53]. Here, the fibrous strips with curved morphology could bear strains of 240% without breaking and achieve better resistance stability under strains.

As shown in Figure 12c, the fibrous strips were assembled on a forefinger, and the electric current was recorded as cyclical bending and release actions proceeding. Figure 12d summarizes the electric current strength changes of the fibrous strips after cyclic bending and release. When the finger was suddenly bent (red circles), the electric current strength decreased sharply, indicating an immediate response to the finger actions. As the angle of the bending finger was not absolutely unanimous, the current strength may differ a little at the bending point of the finger in each cycle. Furthermore, once the finger was completely unfolded, the electric current strength returned sharply to the initial value (blue circles). The current strength could fully recover to its original value at the unfolding point in each cycle. Thus, PS@Fe_3_O_4_ composite nanofibers were realized by magnetic spinning with well-controlled, low-cost, and template-free manners, and the direct-writing fibrous strips are capable of monitoring human motions, indicating potentials as parts of robots and in human–machine interfacing applications. Moreover, attempts should be made to optimize the components and arrangement of direct-writing nanofibers to expand the sensing performance.

## 8. Conclusions

Based on the analysis of the mapping process and evolution mechanism between the design problem, design strategy, and design solution, a problem–strategy–solution (PSS)-based product design solution evolution knowledge service method is proposed, a solution evolution process model based on design iteration is established, and the design of a magnetic melt spinning device is completed, and the following conclusions are drawn.

(1) A model of the solution evolution process based on design iterations is established, and the problem space, strategy space, and solution space are constructed to form three iterative loops of design solution preference, solution strategy adjustment, and problem redefinition. The model establishes a linkage between problem space and solution space with problem solving strategy as the linkage.
(2) Based on the mapping relationship among the design problem, solution strategy, and design solution, a PSS-based product design solution evolution process model is established, and a solution evolution knowledge service model based on design iteration is built from three stages: problem definition, problem solution, and solution evolution, so that each stage of product design is guided by the method and supported by the knowledge provided by the corresponding design strategy.
(3) Taking the design process of the magnetic melt spinning device as an example, under the guidance of the PSS model, the innovative product design solution was formed through the design problem reconstruction, principle innovation, and structure improvement of the spinning device, which verified the feasibility and effectiveness of the method.
(4) In the current study, the magnetic spinning is developed to realize the distinctive deposition of nanofibers with curved structures. The magnetic spinning apparatus is configured by the strong magnetic field and X-Y moving platform, which prepared fibrous strips in curved microstructure with PS@Fe_3_O_4_ nanofiber. Subsequently, conductive polymer composites are constructed into strain sensors, and the currents change sharply in response to the finger bending and release, indicating the capability to monitor human motions. Thus, this study demonstrates a well-controlled and easy-handling strategy of magnetic spinning for direct-writing pattern nanofibers.

However, the knowledge service method for product design solution evolution based on the problem–strategy–solution (PSS) interaction iteration proposed in this paper does not perform well in the face of huge data resources and intelligent decision making. The authors recommend the following future research directions. First, the innovation opportunity identification and evolutionary direction prediction methods based on big data should be further explored. Secondly, an Internet knowledge resource for product innovation design should be constructed. Finally, an effective software tool should be developed to support the application of intelligent evolutionary design methods.

## Data Availability

Not applicable.
